# Quantitative assessment of anatomical change using a virtual proton depth radiograph for adaptive head and neck proton therapy

**DOI:** 10.1120/jacmp.v17i2.5819

**Published:** 2016-03-08

**Authors:** Peng Wang, Lingshu Yin, Yawei Zhang, Maura Kirk, Gang Song, Peter H. Ahn, Alexander Lin, James Gee, Derek Dolney, Timothy D. Solberg, Richard Maughan, James McDonough, Boon‐Keng Kevin Teo

**Affiliations:** ^1^ Department of Radiation Oncology University of Pennsylvania Philadelphia PA; ^2^ Department of Radiology University of Pennsylvania Philadelphia PA USA

**Keywords:** adaptive radiotherapy, treatment planning, proton therapy

## Abstract

The aim of this work is to demonstrate the feasibility of using water‐equivalent thickness (WET) and virtual proton depth radiographs (PDRs) of intensity corrected cone‐beam computed tomography (CBCT) to detect anatomical change and patient setup error to trigger adaptive head and neck proton therapy. The planning CT (pCT) and linear accelerator (linac) equipped CBCTs acquired weekly during treatment of a head and neck patient were used in this study. Deformable image registration (DIR) was used to register each CBCT with the pCT and map Hounsfield units (HUs) from the planning CT (pCT) onto the daily CBCT. The deformed pCT is referred as the corrected CBCT (cCBCT). Two dimensional virtual lateral PDRs were generated using a ray‐tracing technique to project the cumulative WET from a virtual source through the cCBCT and the pCT onto a virtual plane. The PDRs were used to identify anatomic regions with large variations in the proton range between the cCBCT and pCT using a threshold of 3 mm relative difference of WET and 3 mm search radius criteria. The relationship between PDR differences and dose distribution is established. Due to weight change and tumor response during treatment, large variations in WETs were observed in the relative PDRs which corresponded spatially with an increase in the number of failing points within the GTV, especially in the pharynx area. Failing points were also evident near the posterior neck due to setup variations. Differences in PDRs correlated spatially to differences in the distal dose distribution in the beam's eye view. Virtual PDRs generated from volumetric data, such as pCTs or CBCTs, are potentially a useful quantitative tool in proton therapy. PDRs and WET analysis may be used to detect anatomical change from baseline during treatment and trigger further analysis in adaptive proton therapy.

PACS number(s): 87.55‐x, 87.55.‐D, 87.57.Q‐

## I. INTRODUCTION

During fractionated radiation therapy, differences in anatomy are routinely observed on a daily basis. Some examples of these changes include patient weight change, tumor response, patient setup variations, and variations in normal tissue filling and location. The impact on dose distribution arising from anatomic changes may be more pronounced in proton therapy compared to photon therapy,^(1)^ thus it is necessary to find a quick way to identify and quantify these changes and integrate them into the image guidance or adaptive therapy workflow during proton therapy.

Cone‐beam computed tomography (CBCT) facilitates the visualization of the patient's anatomy in the treatment position. In addition to patient alignment, CBCT can also be used to monitor anatomic variations. Compared to multislice fan‐beam CT, the Hounsfield units (HUs) in a CBCT image are highly sensitive to the effects of beam hardening and X‐ray scatter within the patient. Since the proton stopping power is calibrated to the HU,^(2)^ a high degree of HU accuracy is desirable to minimize proton range uncertainty. The HU accuracy of CBCT technology currently is not accurate enough to be used directly for calculation of the water‐equivalent thickness (WET) or resultant proton dose distribution. Changes in scatter conditions due to a 1 cm increase in the radius of a phantom can result in 11% decrease of the HU for water^(3)^ and a corresponding 5% change in calculated WET, which is much larger than the typical proton range uncertainty margin of at least 2.5%−3.5% ^(4)^ used in proton planning.

Deformable image registration provides a means to register the simulation planning CT (pCT) to treatment fraction CBCT.^5^, ^6^, ^7^ The deformed planning CT, which we refer to as the intensity‐corrected CBCT (cCBCT), is geometrically similar to the CBCT but with HUs that are well calibrated for WET calculations. In this work, a diffeomorphic deformable image registration (DIR) toolkit, ANTs (Advanced Normalization Tools),^(8)^ is used to achieve the registration.

Two useful tools for quality assurance in proton therapy are proton radiography^9^, ^10^, ^11^, ^12^ and proton‐computed tomography (proton CT).^13^, ^14^, ^15^, ^16^ Proton radiography permits absolute range verification by measurement of the residual energy of protons that have travelled through a patient. Proton CT offers direct measurements of relative proton stopping powers *in vivo* and avoids the need to convert HUs from X‐ray CT to relative proton stopping power. While these tools are not available for clinical use, a virtual proton radiograph calculated from an X‐ray CT image has other important potential applications in image‐guided proton therapy.

In this work, the utility of virtual proton depth radiographs (PDR), which depict 2D projections of the cumulative WET, is demonstrated. A ray‐tracing technique is used to calculate the WET from a predefined virtual source position through the cCBCT and pCT onto a virtual plane. PDRs are visually similar to a digitally reconstructed radiograph (DRR) used routinely for patient alignment in radiotherapy, but contain information related to cumulative proton stopping power instead of X‐ray attenuation. The difference in PDRs between cCBCT and pCT can be used as a quick and simple online tool for therapists, physicists, and physicians to identify anatomic change from baseline without the need for contouring or dose calculation. Large differences identified during treatment imaging may then be used to trigger further analysis, such as dose calculation off‐line, and determine if adaptive proton therapy is necessary.

## II. MATERIALS AND METHODS

### A. pCT and CBCT data

CBCT for proton therapy is still in development at several institutions and not available clinically at the time of this study, therefore linac CBCTs of one head and neck patient diagnosed with stage IVA malignant neoplasm of the base of tongue and treated with intensity‐modulated radiation therapy was used to demonstrate the proof of principle in this work. The initial volume of the gross tumor volume (GTV) was $50.2\;{\text{cm}}^{3}$. CBCTs were acquired daily for clinical image guidance.

The planning CT (pCT) was acquired in head first supine position on a Philips Gemini TF PET‐CT simulator (Philips Healthcare, Andover, MA). The size of the pCT image was 512×512×208 and resolution was 1.17×1.17×2.0 mm ^3^. During the image registration, the pCT was resliced to the CBCT image resolution and coordinate system.

CBCT images were acquired on a Varian Clinic iX /Trilogy on‐board imaging (OBI) system with the bowtie filter. The X‐ray tube voltage and exposure were 100 kVp and 72 mAs,

respectively. The size of the reconstructed CBCT image was 512×512×64 which corresponds to a resolution of 0.488×0.488×2.5 mm ^3^. Seven CBCT images (one from each week of the treatment) were analyzed.

### B. Deformable image registration algorithm

The image registration was performed using the Advanced Normalization Tools (ANTs), an open source toolbox developed at the Penn Image Computing & Science Lab (PICSL). ANTs utilizes a symmetric image normalization algorithm (SyN) by deforming both images along the shape (diffeomorphism) manifold, guaranteeing that two shapes that are intrinsic to the notion of a geodesic path have the same warping transform regardless of the reference image choice. SyN also guarantees subpixel accuracy for the brain by minimizing interpolation errors.^(8)^ One limitation of this topology‐preserving algorithm is the possible occurrence of topological inconsistencies between the images. These include gas pockets in the rectum or bowel, which may be present in only one image. Since new features cannot be created, the algorithm will produce errors in the registration near these air pockets. This may be circumvented by creating artificial features prior to the DIR to make the images topologically equivalent.^(17)^ For head and neck, larger inaccuracies in the DIR may occur at regions with large differential location of the tissue‐air interface due to effects of swallowing or tongue location, owing to the larger deformations required for matching heterogeneous boundaries.

### C. Registration steps

The pCT (moving image) was first rigidly registered to the CBCTs (fixed images) using mutual information^18^, ^19^ as a metric. Deformable image registration was then applied from the pCT to the CBCTs. In order to achieve fast and accurate deformable image registration, a multimedia technique combined with multiresolution and multimodality was implemented. The original images were down‐sampled by a sequence of factors and the registration was performed iteratively starting from the coarsest level to the finest level. In the multimedia technique, the first stage of deformable image registration was between the bony structures only. The similarity metric used in this stage was mutual information. For the second stage, the deformable registration was applied to the soft tissue only using the mean squared difference metric.^(19)^ The deformed planning CT, which we refer to as the intensity‐corrected CBCT (cCBCT), is geometrically similar to the CBCT but with HUs that are well calibrated for WET calculations.

### D. Assessment of deformable image registration accuracy

The evaluation of DIR accuracy can be roughly categorized into contour‐based or noncontour‐based metrics. The main drawback of the contour‐based evaluation is the relatively high uncertainty of the new contours on the CBCT images with inferior image quality. Therefore, two noncontour‐based metrics were used in this study. The first metric is the cross‐correlation (CC) defined as:

(1) $$CC(x)=\frac{\sum_{i}{\left((I(x_{i})-\mu_{I(x)})(J(x_{i})-\mu_{J(x)})\right)}^{2}}{\sum_{i}{\left(I(x_{i})-\mu_{I(x)}\right)}^{2}\sum_{i}{\left(J(x_{i})-\mu_{J(x)}\right)}^{2}}$$

where *I* and *J* represent the moving and fixed images, *x* is at the center of N×N square window (in two dimensions), *μ* is the mean value within the window centered at *x*, and $x_{i}$ iterates through that window.^(19)^ This method computes a single number after the multi‐iteration calculation and provides a measure of the similarity between the moving and fixed images. For each deformable image registration, the 30 axial slices enclosed between the C2 to C6 vertebrae were chosen for computing the CC value. The average and standard deviation of the CC values of the 30 axial slices are computed.

**Figure 1:**
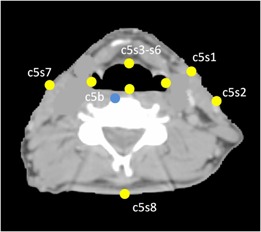
Axial image with landmarks used to validate the deformable image registration. Yellow dots indicate the landmarks located on the boundary between soft tissue and air. Blue dot indicates the landmarks located on the boundary between bone and soft tissue. s1 and s2 are located on the ipsilateral side of tumor.

The second metric is the target registration error (TRE)^(20)^ computed from the geometric distance error of corresponding landmarks between the fixed and registered moving images. Two categories of landmarks were defined: those located either at bone‐to‐tissue boundary or tissue‐to‐air boundary. These landmarks were chosen due to the sensitivity of the cumulative proton WET to heterogeneous tissue, as well as ease of identification on the CT images. For the bone‐to‐tissue category, one landmark on the anterior of each vertebral body of C1 to C5 was picked. For the tissue‐to‐air category, eight landmarks on one CT slice for each cervical vertebrae between C1 to C5 were chosen (Fig. 1).

### E. Polar WET plots

The pCT and cCBCT image intensities for each voxel were converted to relative proton stopping powers using the stoichiometric calibration method,^(2)^ and a ray‐tracing technique was used to determine the water‐equivalent thickness (WET) between a given start and end point. Using the stopping power ratio approximation,^(21)^ the WET through one voxel is the product of the relative stopping power (${\text{RSP}}_{i}$) and path length ($t_{i}$) within that voxel. The total WET of the path is the accumulation of the WET from all voxels traversed by the ray:

(2) $$WET=\sum_{i}t_{i}\times RSP_{i}$$

**Figure 2:**
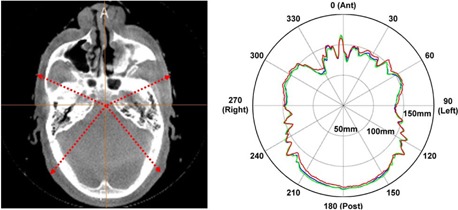
The CT slice (left) used to generate the polar WET plot. The polar WET plots (right). The pCT, cCBCT, and CBCT polar plots are depicted in green, blue, and red, respectively.

Polar WET plots were generated for the assessment of anatomic change on proton range. The 360° polar plot is a measure of the WET from a reference point inside the patient's anatomy to the boundary of the patient's anatomy. Depending on the clinical purpose, the reference point may be chosen to be the image center, the plan isocenter, or a point within the target or organ at risk. In this study, the reference point was chosen to be the image center. Figure 2 illustrates the concept of the polar WET plots for the evaluation of anatomic change. Since there is only minor anatomical change on this image slice between the pCT and the CBCT, the polar plots of the pCT (green) and the cCBCT (blue) overlap almost completely, while the plot using the uncorrected CBCT (red) depict systematic shifts in the WET due to inaccuracies in the HU to stopping power conversion of the original CBCT image.

### F. Virtual proton depth radiography

**Figure 3:**
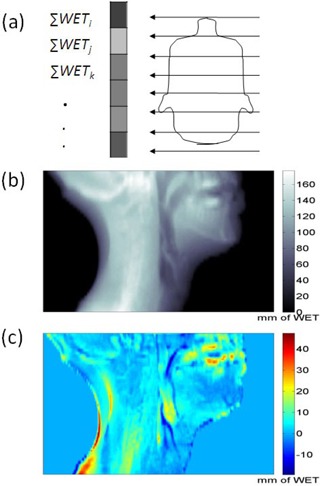
Conceptual illustration (a) of a virtual proton depth radiograph calculated using WETs of patient onto a virtual plane. An example of a PDR (b) and the difference between two PDRs (c). The unit for the scale bar in (b) and (c) is mm.

Lateral proton depth radiographs (PDR) were synthesized from the CT images to depict a two‐dimensional projection of cumulative WETs onto a virtual detector plane, as shown in Fig. 3. PDR differences between the cCBCTs and the reference pCT were evaluated using the distance‐to‐agreement (DTA) technique.(^22,23^) A threshold difference of 3 mm in PDRs and a distance‐to‐agreement (DTA) of 3 mm search radius were used as pass‐fail criteria. The 3 mm search radius permits comparison of PDRs arising from daily setup (3 mm PTV margin) and image registration errors.

An indirect assessment of the DIR accuracy was also performed by evaluating the accuracy of the PDR of a cCBCT from the 5th week by direct comparison with a PDR generated from a replan CT (rCT) performed on the same day. Since the cCBCT should be highly similar to the rCT apart from setup variations, this comparison assesses the accuracy of the deformation of the original pCT to the CBCT in regions where there is significant tumor response and/or weight change.

### G. Comparison between PDR differences and isodose line differences

The impact of WET change on the proton dose distribution was studied by comparing the distal 90% isodose lines of the pCT with those on a forward‐calculated cCBCT. We hypothesize that changes in WET of the patient's anatomy along the beam path can serve as a surrogate for the change of the location of the distal end of the spread‐out Bragg peak (SOBP) leading to possible underdosing of the target and/or overdosing of organ at risk (OAR) in the distal end of the SOBP. In our clinic, the protocol to treat head and neck patients is with a pair of left and right posterior oblique fields. To illustrate the connection between PDR and dose distribution for the field, only the left posterior oblique beam was calculated for this patient. A contour mask was generated using the 90% isodose lines on the pCT and PDRs were calculated on the pCT and the cCBCT using the same mask. The PDR difference image was compared with a map of the WET difference between the distal 90% isodose lines of the pCT and cCBCT (derived from the planning system) in the beam's eye view.

### 433

## III. RESULTS

### A. Deformable image registration results

**Figure 4:**
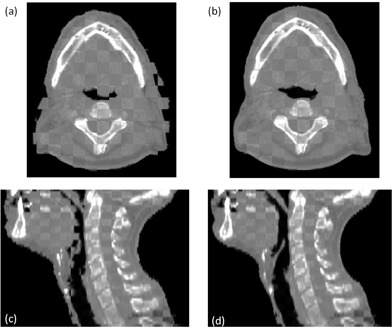
The checker board images of the pCT and CBCT from the second week of the treatment are shown in (a) and (c), respectively. The checker board images of the cCBCT (b) and CBCT (c).

Figure 4 shows a CBCT from the 2nd week of treatment where there is clear evidence of tumor shrinkage and weight loss when compared with the pCT. After DIR, the visual features of the cCBCT (deformed pCT) match very well with the CBCT, especially at the interface of heterogeneous tissue such as the body surface.

### B. Geometric accuracy of deformable image registration

**Figure 5:**
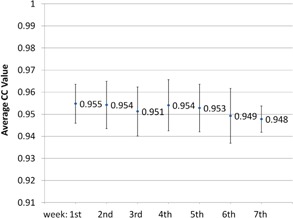
Cross‐correlation results of the similarity between cCBCT and CBCT averaged over 30 axial slices during various weeks of treatment. Error bars indicate ±1 SD.

The CC values between the cCBCT and CBCT calculated with 30 axial slices on different dates are shown graphically in Fig. 5. All CC values were within 0.06 of the maximum value of 1. The average and standard deviation (SD) of the CC value from C2 to C6 (30 axial slices) was 0.95±0.01. There is a small but statistically insignificant decrease in CC values over time which is potentially indicative of larger errors in deformable image registration. This was presumably due to larger anatomic differences between the pCT and the CBCT and, hence, larger extent of deformation required, which may contribute to larger registration errors.

**Figure 6:**
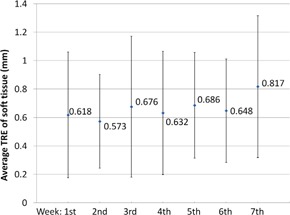
Average and SD of the target registration error between the cCBCT and CBCT measured using soft‐tissue landmarks.

The average and standard deviation of the TRE for bone‐to‐tissue was found to be 0.13±0.25 mm (max: 0.97 mm) which shows superior registration for bony anatomy. The average and standard deviation of the TRE for soft tissue was 0.67±0.43 mm (max: 2.68 mm). The larger distance errors were observed in the oropharynx area due to tumor shrinkage and patient weight loss. The combined average of the TRE versus treatment week is plotted in Fig. 6. These results were slightly better than the 2.8±0.2 mm, 1.0±2.0 mm, and 1.0±0.2 mm errors observed in similar studies that employed different DIR algorithms.^7^, ^24^, ^25^, ^26^

### C. Using PDR difference to predict changes in dose distribution

**Figure 7:**
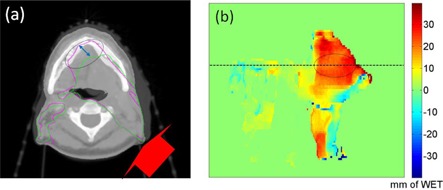
Blended view (a) of the pCT with cCBCT from week 3. The red arrow indicates the direction of the posterior oblique proton beam and the green and magenta contours are the 90% isodose lines for the pCT and cCBCT, respectively. The 2D display (b) of the distal WET difference between the original and new 90% isodose lines in the beam's eye view. The horizontal line indicates the axial slice position of the CT image in (a). Color scale bar is in mm.

**Figure 8:**
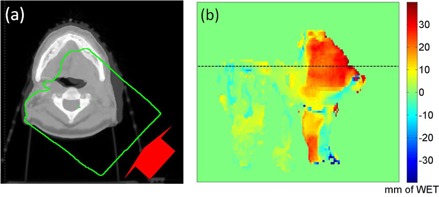
Axial slice (a) indicating the region of PDR calculation (green mask) from the proton source up to the distal 90% dose falloff; (b) beam's eye view PDR difference between the pCT and the cCBCT. The horizontal line indicates the corresponding CT slice in (a). Color scale bar is in mm.

Due to change of the patient's anatomy, the different locations of the 90% isodose lines between the pCT (green) and cCBCT (magenta) is illustrated in Fig 7(a). The change of WET between the distal end of the 90% isodose lines was calculated for all slices in the beam's eye view, as shown in Fig. 7(b). Corresponding PDRs were then calculated in the beam's eye view from the proton source up to the distal edge of the initial 90% isodose line using the contour mask, as shown in Fig 8(a). The change of the PDR between the pCT and cCBCT using this mask is shown in Fig. 8(b). Using a 2 mm WET difference and 2 mm search radius criteria to compare 7, 8, the passing rate of all points was 90.3%. Therefore, the difference in PDRs calculated up to the distal boundary of the original 90% isodose line correlates spatially with change in the distal 90% isodose lines.

### D. Polar WET plots and PDR results

**Figure 9:**
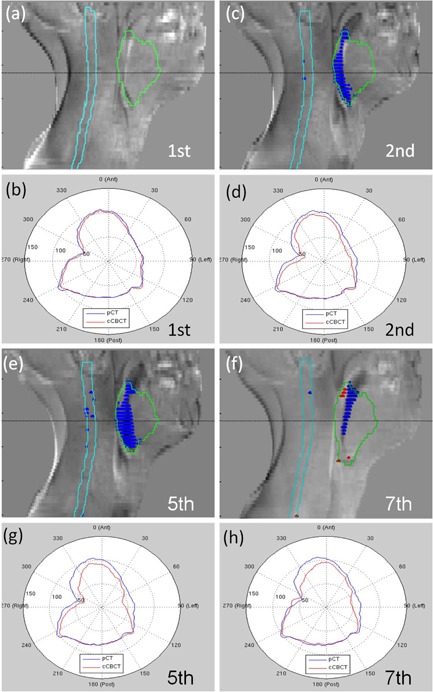
PDR differences and polar WET plots ((a) to (h)) of cCBCT relative to pCT for week 1, 2, 5, and 7. The GTV and spinal cord contours are projected in green and cyan respectively. Blue and red regions indicate points with an accumulated WET difference greater than 3 mm within a 3 mm radius.

PDR differences generated from weekly cCBCTs and the pCT are presented in Fig. 9. The PDRs correspond to cumulative WET from the patient's right to a plane intersecting the midpoint of the spinal cord vertebral body. Differences between the PDRs were mainly found on the boundary of the patient (due to setup variations), pharynx region (due to the change of patient's anatomy), near teeth and high‐density dental implants (image artifacts). PDR differences from the 1st week were small suggesting that the CBCT and pCT were anatomically similar. From weeks 2 through 7, tumor response and patient weight loss was apparent in the CBCTs. Points that exceed 3 mm WET difference and 3 mm search radius within the projected GTV (green) and spinal cord (cyan) are highlighted in blue (potential proton beam overshoot) and red (potential proton beam undershoot). The corresponding polar WET plots of the pCT and cCBCT in one axial slice through the GTV are also presented in Fig. 9.

### E. Comparison of cCBCT and same day rCT PDRs

**Figure 10:**
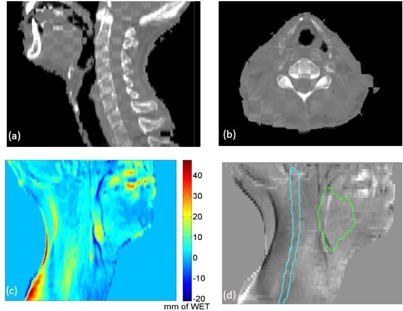
Checker board images of the cCBCT from week 5 and the re‐plan CT acquired on the same day ((a) and (b)). PDR difference (c) with scale bar in mm; (d) no failing points were observed using 3 mm PDR difference and 3 mm search radius within the GTV and cord contours.

Figures 10(a) and (b) show the blended images between the cCBCT from week 5 (used to produce Fig. 9(e)) and the rCT from the same day. Setup errors, such as the skin folding on the left side of the patient's neck, are visible on the patient outline. Figure 10(c) shows the PDR difference generated from the cCBCT and rCT from the same day. Differences between the PDRs were generally small (less than 3 mm), but larger differences were found on the boundary of the patient (due to setup variations), pharynx region (due to motion), and near teeth and high‐density dental implants (image artifacts). All points within the GTV and cord pass the 3 mm WET difference and 3 mm search radius criteria, as shown in Fig. 10(d). This is strong indication that the 3 mm WET difference and 3 mm radius criteria used in the PDR comparison of Fig. 9(e) is sensitive enough to distinguish between real anatomic changes and errors introduced by DIR when generating the cCBCT from the pCT. While it is inevitable that setup differences between the resimulated CT and CBCT from the same day will occur, the cCBCT is a better representation of the patient's anatomy during treatment.

## IV. DISCUSSION

Due to its sensitivity to range uncertainties and anatomical change, proton therapy, especially pencil beam scanning proton therapy requires higher accuracy in image guidance and verification. This requires advanced online volumetric imaging capabilities, such as CT on rail or CBCT system. These imaging systems allow for potential novel applications unique to proton therapy, such as virtual range verification, assessment of anatomical change, and evaluation of plan robustness. Compared to CT on‐rails systems, CBCT is more widely adopted and has played an important role in image‐guided radiation therapy (IGRT) in photon therapy, however it is not widely used in proton therapy yet. Online CBCT systems eliminate the requirement to move patients between treatment and imaging positions. This reduces setup uncertainties and is operationally more efficient than a CT on‐rails system. However, it lacks the HU accuracy of conventional fan‐beam CT scanners. Therefore, implementing CBCT system for proton therapy requires an alternative solution to provide accurate CBCT HU values for proton range computation.

One potential solution is to use deformable image registration (DIR) to warp the pCT onto the CBCT. Our geometric evaluation of DIR accuracy, using the cross‐correlation metric and TRE, shows that this approach is feasible. DIR generally works well in regions with large intensity contrasts, such as bone‐to‐tissue and tissue‐to‐air interfaces. However, in mostly homogeneous soft tissue, the geometric accuracy of DIR may have larger errors that impact other radiation therapy applications, such as dose accumulation and contour propagation. WET calculations are less sensitive to DIR errors within low‐contrast soft tissue since the cumulative variation of relative stopping power error along the beam path is small. It is, however, more sensitive to geometric errors at heterogeneous interfaces since variations in the relative stopping power are larger. An alternative approach to the HU inaccuracy problem without using DIR is to use tissue class (air, soft tissue, lung, and bone) assignment of relative proton stopping power for calculating WETs of both the CBCT and pCT. This simplified approach may be less accurate for WET computation, but has the advantage that the PDRs can be computed quickly. Regardless of the approach, techniques to experimentally validate calculated WET values include proton radiography,^(12)^ proton range probe,^(27)^ or an *in vivo* dosimetry method.^(28)^

Volumetric imaging with accurate HUs, enables new analysis techniques to be developed for proton therapy. As a proof of concept, lateral PDRs of a head and neck patient who demonstrated considerable anatomic change over the course of treatment were evaluated. In a multifield proton treatment plan, PDRs may be generated from the beam direction onto a plane distal to the maximum proton range. Regions with large differences in the accumulated WETs will highlight potential proton beam range deviations from the plan. As we demonstrated in Figs. 7 and 8, differences in the PDR for each field translate to shifts in the corresponding distal isodose lines and may be used as a surrogate to identify potential dosimetric deviations. By defining action thresholds based on clinical evidence and treatment planning protocols, these tools may be used either offline or online to efficiently and objectively assess changes in the patient's anatomy and setup variations, and make clinical decisions regarding the need for adaptive proton therapy (replanning). The WET and PDR analysis presented in this work to identify anatomical change requires only the patient body contour on the CBCT and is a practical method for online assessment of the CBCT during patient setup and treatment. For proton centers with an in‐room CT scanner, the additional step of image intensity correction is not necessary.

**Figure 11:**
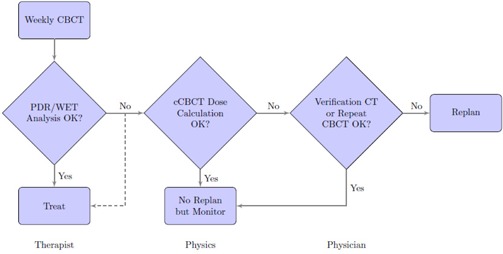
Proposed workflow of adaptive proton therapy using PDR and WET analysis to determine if further action needs to be performed.

As CBCT for proton therapy becomes more widely available, it is expected to have a larger impact in adaptive therapy compared to photon therapy. Advanced analysis tools to evaluate the distal range of the proton beam, polar WET maps, tracking PDR changes as well as the potential to compute the “dose of the day”^7^, ^26^ using the cCBCT, are some applications that may be developed using volumetric imaging to leverage the maximum benefit from the unique properties of proton dose distribution. If this analysis can be automated, it may be possible to use CBCT to trigger further action such as dose calculation on the cCBCT or resimulation and replanning. Such a workflow is depicted in Fig. 11, whereby dose calculation on the cCBCT may be necessary only if anatomical change from baseline above a threshold is detected using the PDR tool. Dose calculation and recontouring may then be performed offline, and the need for a verification CT to verify the cCBCT dose distribution is determined. It will also be important to be able to differentiate random positional change with systematic anatomic differences as treatment proceeds. This information may inform how treatment margins for proton therapy are determined in the future.

## V. CONCLUSIONS

We have presented the concept of using an HU‐corrected CBCT to generate polar WET plot and virtual PDRs to assess the impact of anatomic change in proton therapy. Differences observed in PDRs correlated with patient anatomy change and the distal dose distribution. The virtual PDRs may be used as a tool for assessing potential proton range deviations from each beam direction and enable online decisions about the treatment plan to be made and trigger adaptive proton therapy. The feasibility of using DIR to map the HU value from a pCT to a CBCT was demonstrated. The average and standard deviation of the distance error for tissue‐to‐air was 0.67±0.43 mm (max: 2.68 mm) and 0.13±0.25 mm (max: 0.97 mm) for bone‐to‐tissue.

## COPYRIGHT

This work is licensed under a Creative Commons Attribution 4.0 International License.
